# Retrospective study of inflammatory markers in high-grade cervical lesions

**DOI:** 10.1590/1806-9282.20260706

**Published:** 2026-10-05

**Authors:** Pınar Yıldız, Güneş Topçu, Gerçek Aydın, Asiye Uzun

**Affiliations:** 1 Academic Hospital, Department of Obstetrics and Gynecology – Istanbul, Turkey.; 2 Bahçelievler Medipol University Hospital, Department of Obstetrics and Gynecology – Istanbul, Turkey.; 3 Istanbul Health and Technology University, Department of Obstetrics and Gynecology – Istanbul, Turkey.

**Keywords:** Cervical intraepithelial neoplasia, Human papillomavirus, Papanicolaou test, Inflammation

## Abstract

**OBJECTIVE:**

The aim of this study was to evaluate the predictive value of systemic inflammation markers for abnormal cervical screening results and high-grade cervical lesions in women participating in a human papillomavirus- and cytology-based screening program, thereby determining colposcopy indications more accurately to reduce unnecessary colposcopy requests and prevent overtreatment.

**METHODS:**

A total of 2,758 women who underwent cervical cancer screening between 2020 and 2025 were included in this retrospective cross-sectional study. Abnormal screening results were defined as abnormal cytology (≥Atypical squamous cells of undetermined significance [ASC-US]) and/or high-risk human papillomavirus positivity. Hematological parameters measured within 1 month prior to screening were used to calculate neutrophil-to-lymphocyte ratio, platelet-to-lymphocyte ratio, and systemic immune-inflammation index. Continuous variables were evaluated using nonparametric tests, and the independent predictors of abnormal screening results and high-grade lesions were identified using logistic regression models.

**RESULTS:**

Neutrophil-to-lymphocyte ratio, platelet-to-lymphocyte ratio, and systemic immune-inflammation index values were significantly higher in the abnormal smear groups and in human papillomavirus -positive patients (all p<0.001). Neutrophil-to-lymphocyte ratio displayed the highest diagnostic performance for high-grade lesions (area under the curve=0.871; cutoff value >4.41, sensitivity 66.7%, specificity 99.2%), followed by systemic immune-inflammation index (area under the curve=0.847), while platelet-to-lymphocyte ratio showed lower specificity (area under the curve=0.741).

**CONCLUSION:**

In conclusion, our findings suggest that inflammatory markers are associated with abnormal cervical screening results. To determine the significance and clinical utility of serum inflammatory markers, multicenter clinical trials involving larger patient cohorts are needed.

## INTRODUCTION

Cervical cancer is a type of malignant tumor affecting the female reproductive system; it is the most common gynecological cancer in developing countries and accounts for the fourth most common type of cancer worldwide^1^ Global Cancer Statistics (GLOBOCAN 2022) data indicate that 662,301 women were diagnosed with cervical cancer in 2022, and an estimated 348,874 died from the disease^2^. As cancer screening programs have become more widely available, it is known that the rate of diagnosed cervical intraepithelial neoplasia (CIN) has increased. In contrast, the incidence of advanced-stage cancer has decreased^3^. Early detection plays an important role in the prevention and treatment of cervical cancer^4^. During clinical practice, women who receive abnormal screening results should be evaluated with a colposcopy and biopsy to detect precancerous lesions and cervical cancer^5^.

Human papillomavirus infection is a major risk factor associated with the development of cancer and can potentially lead to CIN. This infection is relatively common and affects approximately 90% of people during their lifetime, potentially leading to serious health issues. There are more than 200 genotypes of HPV. While the majority of them are not associated with complications and resolve spontaneously, some types are considered high-risk and can lead to cancer^6,7^.

In recent studies, the potential value of hematological markers in the early diagnosis and prognosis of cancers has been recognized. It is considered that inflammatory parameters such as the neutrophil-to-lymphocyte ratio (NLR), platelet-to-lymphocyte ratio (PLR), and systemic immune-inflammation index (SII) are correlated with tumor cell formation, progression, and prognosis^8^. The cytokines, vascular endothelial growth factor, and chemokines produced by neutrophils are involved in tumor growth, angiogenesis, and metastasis. In the same way, platelets stimulate tumor cell proliferation and help these cells evade immune control, thereby promoting tumor spread^9^. The is a novel marker of inflammation and a useful biomarker for assessing the prognosis of various cancers; it is calculated using the formula: platelet count × neutrophil count/lymphocyte count^10,11^. Several inflammatory markers in the blood are linked to the formation and development of tumors in various types of cancer^12,13^. In our study, 2,758 patients who had undergone smear tests were retrospectively analyzed to ensure accurate patient assessment, improve screening effectiveness, prevent unnecessary colposcopy and biopsy referrals, and reduce overtreatment to identify more effective alternative biomarkers for predicting cervical lesions and establishing more appropriate clinical criteria for colposcopy.

## METHODS

### Study design and setting

This study was designed and reported in accordance with the STROBE Statement. It was conducted as a single-center, retrospective descriptive study at the Department of Obstetrics and Gynecology of a tertiary care university hospital in Istanbul, Türkiye.

### Study population and sampling strategy

The study population consisted of women who presented to our outpatient clinic, had a complete blood count within 1 month, and underwent cervical cytology screening between January 1, 2020, and January 1, 2025. During the study period, a total of 2,758 pap smear tests and blood tests were performed. All women aged 21–65 years who were not pregnant, had no history of treatment for cervical lesions, had a pap smear and/or HPV testing and a blood test within the defined period, and had complete medical records were included. Patients under 21 or over 65 years of age, those with organ failure, people with an active infection or malignant neoplastic diseases, those who had been treated with antibiotics before testing, those taking medication impacting inflammatory markers, and pregnant women were excluded. A total of 82 cases were excluded due to not meeting the inclusion criteria and/or having incomplete data.

### Research questions

The study aimed to evaluate effective alternative biomarkers for predicting cervical lesions and to assess their impact on clinical processes in determining the requirement for colposcopy.

### Data collection and variables

Data were retrospectively obtained from the hospital’s Electronic Health Record (EHR) system. The following variables were recorded: age, parity, pap smear and HPV test results, PLR, NLR, and SII values based on the complete blood count results. All data were anonymized before analysis.

### Definition of exposure and outcome measures

Exposure variables were defined as hematological inflammation parameters, including NLR, PLR, and SII, derived from complete blood count measurements obtained within 1 month preceding cervical cancer screening.

NLR=neutrophil count/lymphocyte count

PLR=platelet count/lymphocyte count

SII=(platelet × neutrophil)/lymphocyte

We defined the primary outcome as the presence of an abnormal cervical screening result. Abnormal cytology (≥ASC-US) and/or high-risk HPV positivity were considered to constitute this outcome. Secondary outcomes consisted of the presence of high-grade cytological abnormalities (≥HSIL), evaluation of the relationship between inflammatory indicators and the presence of HPV, and investigation of the performance of hematological inflammation indices in the prediction of abnormal screening results.

### Follow-up

No follow-up was conducted because of the study’s retrospective design.

### Handling of missing data

Cases with incomplete or missing data were excluded from the study.

### Statistical analysis

Statistical analyses were performed using SPSS version 27. Quantitative variables were expressed as mean, standard deviation, median, minimum, and maximum, while qualitative variables were presented as frequencies and percentages. The Fisher-Freeman-Halton exact test was used to compare qualitative data, and p<0.01 was considered statistically significant.

### Ethical considerations

Ethical approval was obtained from the Institutional Ethics Committee (Approval No: 5163; Date: November 11, 2025). The study was conducted in accordance with the ethical principles of the Declaration of Helsinki and the ethical standards of the country of origin. The authors declare no conflicts of interest.

## RESULTS

A total of 2,758 patients were included in the study. Continuous variables deviated from a normal distribution, as expected given the large sample size (Shapiro-Wilk, p<0.001), and were reported as medians (IQR); categorical variables were presented as frequencies and percentages.

The median age of the study population was 37 [30–42] years, and 61.2% of the women (n=1,689) were multiparous. Based on the smear results, 66.8% of patients (n=1,841) had benign cytology, 18.2% (n=503) were ASC-US, and 11.3% (n=312) were LSIL. High-grade lesions (ASC-H, HSIL, AGC, AIS, and SCC/AdenoCa) accounted for 3.7% (n=102) of all cases. HPV positivity was observed in 20.8% of patients (n=573), and the most common subtype was HPV 16 (7.7%) (Table 1).

**Table 1:** Demographic, clinical, and laboratory characteristics of the study population.

Variable	n/median (IQR)	%/(min–max)	Shapiro-Wilk p-value
Demographic characteristics			
Number of patients	2,758	–	–
Age (years)	37 (30–42)	21–53	<0.001
Parity—nulliparous	1,069	38.8	–
Parity—multiparous	1,689	61.2	–
Hematological parameters			
NLR	2.04 (1.61–2.53)	0.30–15.72	<0.001
PLR	125.9 (101.4–156.9)	32.4–1,200.0	<0.001
SII (neutrophil × platelet/lymphocyte)	554.1 (414.1–741.6)	88.3–7,104.0	<0.001
Smear results			
Benign	1,841	66.8	–
ASC-US	503	18.2	–
LSIL	312	11.3	–
ASC-H	40	1.5	–
HSIL (CIN2–3)	48	1.7	–
AGC/AIS/SCC–adenocarcinoma	14	0.5	–

Continuous variables are presented as median (IQR, Q1–Q3). All continuous variables showed non-normal distribution (Shapiro-Wilk p<0.001). Categorical variables are expressed as n (%). IQR: interquartile range; NLR: neutrophil-to-lymphocyte ratio; PLR: platelet-to-lymphocyte ratio; SII: systemic immune-inflammation index (neutrophil × platelet/lymphocyte); LSIL: low-grade squamous intraepithelial lesion; ASC-H: atypical squamous cells, cannot exclude HSIL; HSIL: high-grade squamous intraepithelial lesion; CIN2–3: cervical intraepithelial neoplasia grades 2–3; AGC: atypical glandular cells; AIS: adenocarcinoma in situ; SCC: squamous cell carcinoma; ASC-US: atypical squamous cells of undetermined significance.

NLR, PLR, and SII values were significantly increased in line with cytological grade and were higher in groups with abnormal smear results compared to benign cases (all p<0.001). These markers were also elevated in HPV-positive patients, who had a significantly lower mean age. HPV positivity varied significantly among pap smear groups [χ^2^(7)=873.17, p<0.001]; this rate increased with the severity of cytology, rising from 5.2% in benign cases to 49.9% in ASC-US, 72.9% in HSIL, and 100% in high-grade lesions.

Spearman’s correlation analysis revealed moderate-to-strong positive correlations for NLR with smear results, colposcopy findings, and HPV positivity (r=0.514–0.665; all p<0.001). For SII, similar but weaker associations were found (r=0.425–0.554). PLR exhibited weak correlations (r=0.261–0.329). Age showed weak and negative correlations with NLR and SII (Table 2).

**Table 2:** Correlation of neutrophil-to-lymphocyte ratio, platelet-to-lymphocyte ratio, and systemic immune-inflammation index with clinical variables.

Marker	Age r (p)	Smear result r (p)	Colposcopy r (p)	HPV positivity r (p)
NLR	-0.048 (0.011)*	0.514 (<0.001)***	0.572 (<0.001)***	0.665 (<0.001)***
PLR	-0.034 (0.078) ns	0.261 (<0.001)***	0.277 (<0.001)***	0.329 (<0.001)***
SII	-0.038 (0.048)*	0.425 (<0.001)***	0.477 (<0.001)***	0.554 (<0.001)***

Spearman correlation analysis.

*p<0.05

***p<0.001; ns: not significant. NLR: neutrophil-to-lymphocyte ratio; PLR: platelet-to-lymphocyte ratio; SII: systemic immune-inflammation index.

In univariate analysis, NLR, PLR, SII, and HPV positivity proved to be significant predictors of each outcome (all p<0.001). In multivariate analysis, however, the significance of PLR was reduced. While NLR (OR 3.627) and HPV positivity (OR 1.755) were independent predictors of abnormal smear results, NLR (OR 2.245), SII (OR 1.439), and HPV positivity (OR 1.957) independently predicted advanced lesions (all p<0.001).

The ROC analysis indicated that NLR demonstrated the highest diagnostic performance in identifying high-grade lesions (AUC=0.871); the optimal cutoff value was >4.41 (sensitivity 66.7%, specificity 99.2%). SII also demonstrated acceptable performance (AUC=0.847), whereas PLR showed lower accuracy (AUC=0.741). Since the cutoff values were obtained from the same dataset, external validation is required (Table 3).

**Table 3:** ROC analysis of neutrophil-to-lymphocyte ratio, platelet-to-lymphocyte ratio, and systemic immune-inflammation index for the diagnosis of high-grade cervical lesions.

Marker	AUC (95%CI)	Optimal cut-off	Sensitivity (%)	Specificity (%)	PPV (%)	NPV (%)
NLR	0.871 (0.827–0.916)	>4.41	66.7	99.2	76.4	98.7
SII	0.847 (0.800–0.895)	>818.65	74.5	83.1	14.5	98.8
PLR	0.741 (0.685–0.797)	>133.94	81.4	58.8	7.1	98.8

Optimal cutoff values were determined using the Youden index. AUC confidence intervals were calculated by the DeLong method. An AUC >0.80 was considered to indicate good discriminatory performance. PPV: positive predictive value; NPV: negative predictive value; NLR: neutrophil-to-lymphocyte ratio; PLR: platelet-to-lymphocyte ratio; SII: systemic immune-inflammation index; CI: confidence interval; AUC: area under the curve

## DISCUSSION

This study reveals that systemic inflammatory markers show a statistically significant correlation with the severity of cervical cytopathology and HPV positivity in a large screening cohort. Our key finding demonstrates a progressive increase in these markers as cervical lesions progress from benign to high grade. Specifically, the NLR emerged as the strongest independently predictive marker for high-grade lesions (HSIL+), with a remarkable specificity of 99.2% at a cutoff value of 4.41.

The linear progression of HPV positivity—ranging from 5.2% in the benign group to 100% in high-grade lesions—highlights the critical role HPV performs in the development of cervical cancer. This tendency is consistent with the established understanding that high-risk HPV types promote a chronic inflammatory environment prone to neoplastic transformation^14^. Our results suggest that this inflammation is not limited to the local area, but appears to be systemic, as indicated by elevated hematological indices. The strong correlation between HPV positivity and NLR (r=0.665) suggests that the systemic inflammatory response is linked to viral load or the persistence of high-risk HPV subtypes. This situation is consistent with recent findings regarding the complementary role of HPV status in cytological smear reporting^15^. Additionally, in a study carried out by Tóth et al. involving 395 patients, the NLR was observed to be significantly associated with HPV DNA positivity and p16 expression; the NLR was found to be higher in HPV-positive patients. But its diagnostic performance remained moderate (AUC≈0.61)^16^.

In a 2024 study by Qin and Zhang evaluating 324 participants, it was found that although NLR, PLR, and SII levels were significantly higher in patients with cervical lesions, the predictive power of these markers alone was restricted, and their AUC values were relatively low, which indicates that the clinical utility of these markers as triage biomarkers is limited. Compared with other studies, our study achieved an AUC of 0.871 for NLR, which is statistically significantly higher than previously reported values in the literature, including the AUC of 0.569 reported by Qin and Zhang^17^. This inconsistency may be related to our larger sample size (n=2,758) and the inclusion of a wider range of cytological abnormalities in the study.

Atlıhan et al. noted that although both NLR and PLR levels tended to increase with the severity of CIN, only NLR showed a statistically significant association, whereas PLR was not an independent predictor^18^. The lower specificity of the PLR may be attributed to the fact that platelet count is influenced by multiple factors. Consequently, the NLR appears to be a more reliable biomarker for cervical lesions. While the PLR was found to be meaningful in the univariate analysis, it likely became insignificant after adjustment due to multicollinearity with the NLR and SII. In contrast, NLR was a significant independent predictor in all models; this confirms its clinical value as a simpler and more reliable parameter compared to PLR or SII.

Our findings have significant clinical value. Given the NLR’s high specificity (99.2%) at a cutoff >4.41, this indicator can serve as a meaningful “diagnostic verification” tool in triage procedures. In situations where resources are limited or as a supplement to primary HPV/cytology screening, the NLR can help determine which patients should be referred for colposcopy, potentially reducing unnecessary procedures while ensuring that high-risk patients receive prompt treatment. The availability and cost-effectiveness of complete blood counts enhance the feasibility of incorporating these markers into routine clinical practice^19^.

Despite the strengths of this study, certain limitations must also be considered. First, the retrospective nature of this study complicates the identification of a definitive causal relationship between systemic inflammation and the progression of cervical lesions. The small sample sizes in rare high-risk groups (AGC, AIS, SCC/adenocarcinoma) limit the generalizability of the findings. Also, although we have determined optimal threshold values, they were derived from the same data set; therefore, an external validation study in independent cohorts is required to verify their diagnostic performance^17^.

## CONCLUSION

In conclusion, markers of systemic inflammation are associated with abnormal cervical screening results, but their clinical utility varies. Among these, NLR appears to be a reliable independent predictor of abnormal cytology and high-grade lesions, while PLR lost its significance after adjusting for covariates, and SII did not demonstrate superior performance. While these findings suggest that inflammation may play a role in cervical carcinogenesis and HPV persistence, they indicate that NLR is the most practical biomarker. Given their limited discriminatory ability, however, these markers should be considered complementary rather than primary screening tools. Future multicenter prospective studies are needed to validate these findings.

## Data Availability

The datasets generated and/or analyzed during the current study are available from the corresponding author upon reasonable request.
